# *mCherry* contains a fluorescent protein isoform that interferes with its reporter function

**DOI:** 10.3389/fbioe.2022.892138

**Published:** 2022-08-09

**Authors:** Maxime Fages-Lartaud, Lisa Tietze, Florence Elie, Rahmi Lale, Martin Frank Hohmann-Marriott

**Affiliations:** ^1^ Department of Biotechnology, Norwegian University of Science and Technology, Trondheim, Norway; ^2^ United Scientists CORE (Limited), Dunedin, New Zealand

**Keywords:** *mCherry*, fluorescent protein, reporter gene, alternative translation initiation site, gene expression, fusion protein

## Abstract

Fluorescent proteins are essential reporters in cell and molecular biology. Here, we found that red-fluorescent proteins possess an alternative translation initiation site that produces a short functional protein isoform in both prokaryotes and eukaryotes. The short isoform creates significant background fluorescence that biases the outcome of expression studies. In this study, we identified the short protein isoform, traced its origin, and determined the extent of the issue within the family of red fluorescent protein. Our analysis showed that the short isoform defect of the red fluorescent protein family may affect the interpretation of many published studies. We provided a re-engineered *mCherry* variant that lacks background expression as an improved tool for imaging and protein expression studies.

## Introduction

The discovery of fluorescent proteins has played a major role in unraveling details of cellular functions (Day and Davidson, 2009; Chudakov et al., 2010). Fluorescent proteins display structural similarities, such as a fully amino acid-encoded chromophore present on an α-helix that is tightly packed into an 11-stranded ß-barrel (Ranganathan et al., 2000; Wachter et al., 2010). The chromophore is created by the autocatalytic cyclization of an amino acid triad (Gross et al., 2000; Shu et al., 2006; Wachter et al., 2010). The discovery of DsRed expanded the color range of fluorescent proteins to include red wavelengths (Matz et al., 1999). The natural versions of fluorescent proteins have been subject to a multitude of modifications to obtain different colors (Labas et al., 2002; Mishin et al., 2008; Wachter et al., 2010; Subach and Verkhusha, 2012) and improve their properties, such as solubility, maturation, stability, quantum yield, monomeric state, or the ability to uptake a fusion partner (Lauff and Hofer, 1984; Bevis and Glick, 2002; Campbell et al., 2002; Shaner et al., 2004; Rodriguez et al., 2017). This diversity of engineered fluorescent proteins have emerged as invaluable tools for molecular and cell biology, as they are excellent reporters for gene expression and subcellular protein localization in various biological systems (Day and Davidson, 2009; Chudakov et al., 2010; Rodriguez et al., 2017).

In a previous study, we used *mCherry* as one of the reporters for the development of a universal gene expression method that employs 200 random nucleotides as regulatory sequence to drive coding sequence expression (Lale et al., 1101). The efficiency of the method is gene- and context-dependent, but it usually yields between 30% and 40% of successful protein expression in *Escherichia coli* (Lale et al., 1101). However, when *mCherry* is used as a reporter, we observe fluorescence in about 65% of *E. coli* clones (Supplementary Figure S1). In addition, a large-scale analysis of the transcription start sites of these clones showed a nonnegligible fraction of leaderless mRNA sequences (Lale et al., 1101). Unsettled by these high proportions, our suspicion turned to the reporter sequence itself. We hypothesized that internal Shine–Dalgarno (SD) sequences within the reporter sequence followed by methionine codons just downstream the actual start codon could result in the expression of a shorter yet still functional version of *mCherry*. This constitutes an equivalent to Russell’s paradox in molecular biology, more specifically in relation with the red fluorescent protein *mCherry* (*see footer): *mCherry* does not contain itself; however, we suspected that it does (Supplementary Figure S2).

A fraction of eukaryotic and prokaryotic genes possess alternative translation initiation sites (ATIS) that lead to the production of different isoforms of a functional protein from a unique mRNA (Wegrzyn et al., 2008; Fritsch et al., 2012; Wan and Qian, 2014; Nakahigashi et al., 2016). The N-terminal sequence variation between protein isoforms can be the target of posttranslational regulation (Trulley et al., 2019), affect protein functionality (Ozin et al., 2001; Bernier et al., 2018), and even direct subcellular localization (Chabregas et al., 2003). The presence of an ATIS in *mCherry* greatly affects its function as a reporter and the outcome of experiments. A shorter version of *mCherry* contained in itself creates a nonnegligible background fluorescence, disturbing the results of gene expression and protein localization studies. For example, a genetic construct encoding a fusion protein composed of a C-terminal *mCherry* presents a risk of producing an independent, fusion-less *mCherry* protein, which interferes with protein localization. Likewise, studies using *mCherry* as reporter for gene expression would yield biased results because translation of the short *mCherry* isoform is included in the reporter gene sequence. As *mCherry* is widely used, the postulated interference may affect many studies, including our own work. However, like Frege, we believe that the advancement of knowledge deserves our full dedication. Therefore, we investigated *mCherry* expression in detail to uncover the source of the issue with the goal of providing solutions that eliminate the defectof translation initiation.

## Results and discussion

### Identification of the shorter protein isoform of *mCherry*

**FIGURE 1:**
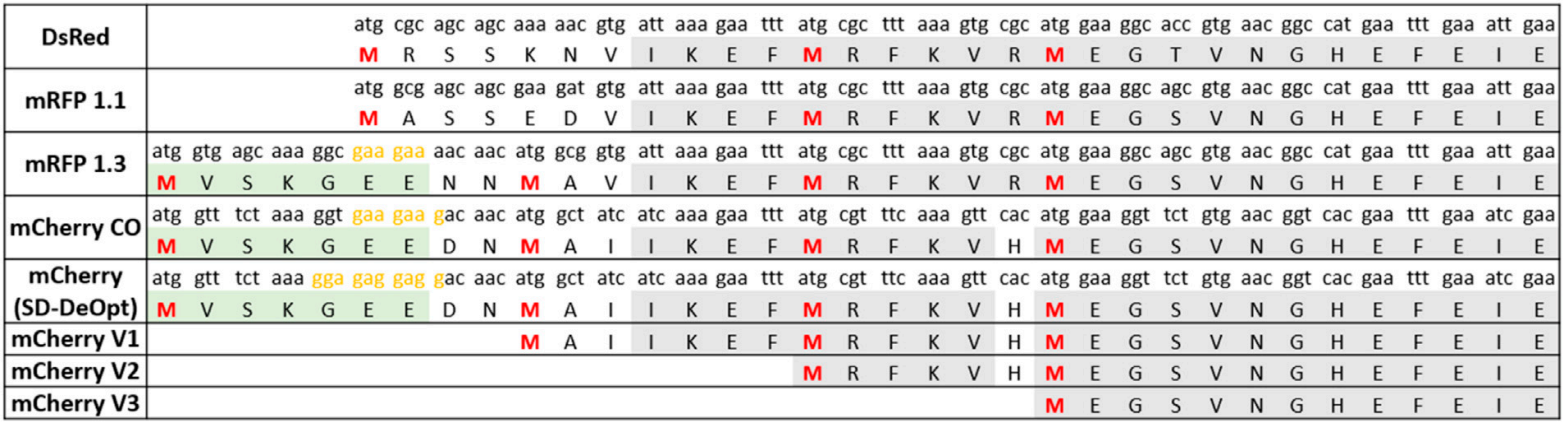
Amino acid sequence alignment of key engineering steps of red fluorescent proteins derived from DsRed. The isoform-causing N-terminal extension occurred in the engineering step from mRFP1.1 to mRFP1.3 due to of the addition of the eGFP fragment (in green) and the linker NNMA. The sequences of the short *mCherry* versions (V1, V2, and V3) used to determine the shortest functional isoform are also presented. Furthermore, the sequence of the “Shine–Dalgarno (SD)-deoptimized” version of *mCherry* is shown. Methionines potentially serving as start codons are shown in red (M1, M10, M17, and M23). SD-like sequences are presented in yellow. A codon usage variant of *mCherry* was created, named SD-deoptimized, which refers to the strongest SD sequence possible upstream M10 from codon usage variations that would increase background expression. The codon usage of each protein is indicated above each amino acid. The conserved amino acid sequence is presented in gray.

Our previous experiments with *mCherry* as a reporter gene (Lale et al., 1101) led us to suspect the presence of a shorter protein isoform of *mCherry*. We first analyzed the sequence of the codon-optimized version of *mCherry* and noticed three methionine residues in a relatively close proximity from the annotated start codon (Figure 1). In addition, we recognized an SD-like sequence ranging from −12 to −6 nucleotides upstream of the first internal methionine residue (Figure 1). This led us to hypothesize that a short functional isoform of *mCherry* is produced from one of the three downstream methionines and not from an alternative start codon.

**FIGURE 2:**
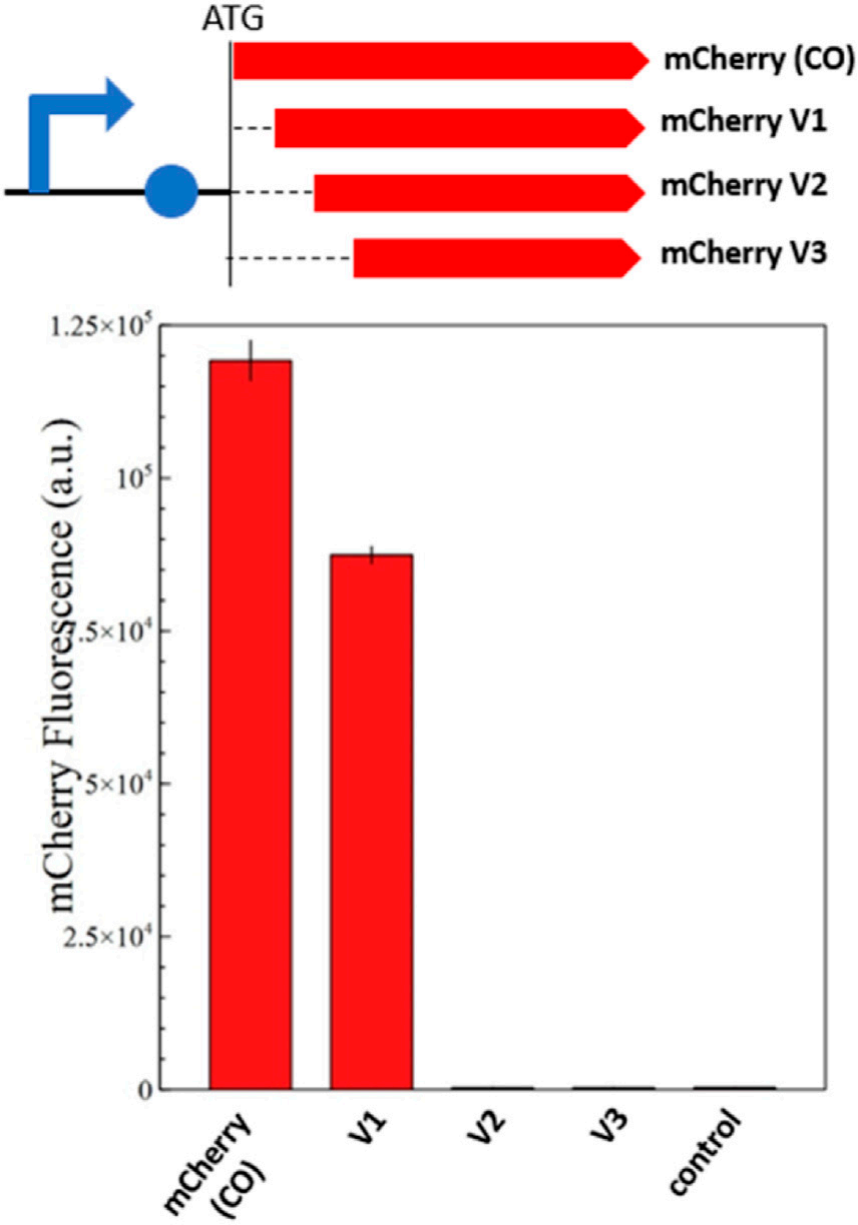
Fluorescence measurements of *mCherry* and its shorter isoforms (V1, V2, and V3). The genetic map of each constitutively expressed *mCherry* version is shown above the fluorescence quantification graph. The graph shows the *in vivo* fluorescence of the different *mCherry* versions in *E. coli* normalized by OD_600_. A plasmid without *mCherry* is used as a negative control. Four biological replicates were made for each *mCherry* version, and the respective standard deviation is presented with black error bars. Just *mCherry* V1 produces a functional protein isoform that retains 73% of the original fluorescence; whereas V2 and V3 are not fluorescent.

To determine which of these methionine residues function as an ATIS that still renders a functional *mCherry* protein, we designed three versions of *mCherry* (V1, V2, and V3), with increasing N-terminal truncations (Figure 1). Each version of *mCherry* was expressed in *E. coli* with a constitutive promoter/5′-UTR. The fluorescence measurements of the different *mCherry* versions are presented in Figure 2. The smallest truncation (V1) retains 73% of the fluorescence intensity of the original codon-optimized version (*mCherry-CO*); whereas, the other versions (V2 and V3) do not show any fluorescence. In addition, proteomics analysis of the red fluorescent protein *mCherry* V1 confirmed that the first 10 amino acids were absent (Supplementary Figure S3), although the fragment between M10 and M17 could not be detected due to the short length of the digested fragments. This is conclusive evidence that the *mCherry* gene produces a short functional protein isoform starting at the methionine in position 10 of the amino acid sequence.

### Phylogenic analysis reveals the origin and extent of the problem

**FIGURE 3:**
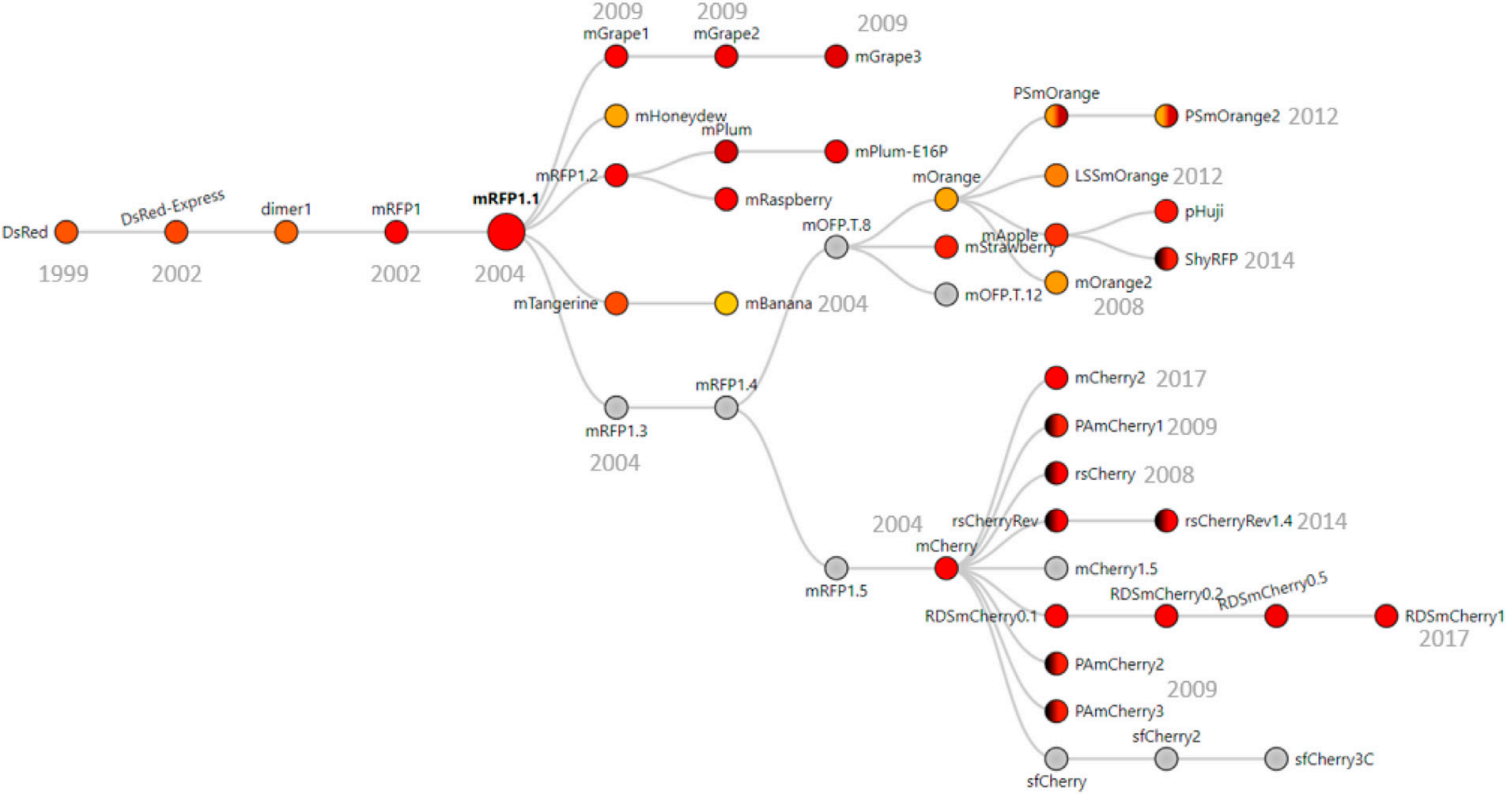
Phylogenic tree of DsRed-derived fluorescent proteins. Figure adapted from FPbase (Lambert, 2019) with their permission. The phylogenic tree shows all the fluorescent proteins derived from mRFP1.3 that contains the problem-causing N-terminal extension. Underlined proteins are derived from mRPF1.1 that also contain the extension. Color in circles corresponds to the emission wavelength(s) of the protein. Dark hemicycles represent an off-state of the protein. Gray indicates that the emission maximum was not entered in the database. The year in which the proteins were engineered is indicated in gray below/next to the corresponding protein.

Because most red fluorescent proteins originate from the modifications of DsRed (Matz et al., 1999), we imagined that the dual-isoform issue of *mCherry* could affect other members of the red fluorescent protein family. We performed protein sequence alignments to determine the extent of the issue across DsRed-derived fluorescent proteins (Figure 1). We were able to trace back the apparition of the second isoform to the engineering of mRFP1.3 (Figure 3).

DsRed was modified into mRFP1 to overcome obligate tetramerization, improve protein maturation, and modify the excitation/emission wavelength couple (Campbell et al., 2002). Then, mRFP1 was further modified into mRFP1.1 by the notable Q66M substitution in the chromophore to improve parameters such as photostability, quantum yield, and extinction coefficient (Shaner et al., 2004). During the same round of modifications, in an attempt to improve the poor N-terminal fusion properties of mRFP1.1, the initial residues of eGFP (MVSKGEE) followed by the linker (NNMA) were added to mRFP1.1 to yield mRFP1.3^12^. This manipulation is responsible for the apparition of the short *mCherry* isoform. Indeed, the linker (NNMA) provides the alternative methionine start codon, and the eGFP fragment offers an SD-like sequence for ribosome entry (Figure 1).

The phylogenic overview of red fluorescent proteins (Figure 3) shows all the proteins engineered from mRFP1.3 that are affected by the dual-isoform issue. In addition to the mRFP1.3-derived proteins, mBanana, mGrape2, and mGrape3 also received the N-terminal extension described above. The problem becomes even more concerning because it affects all commonly used variants in the red color spectrum. Furthermore, the timeline of engineering is important. As mRFP1.3 and *mCherry* were created in 2004, this entails that the results from a large number of publications using red fluorescent proteins since 2004 may have been affected by this issue.

### Short *mCherry* isoform is also expressed both in prokaryotes and eukaryotes

In prokaryotes, translation initiation shares quasiuniversal principles (Nakagawa et al., 2010; Rodnina, 2018), with some variations in the SD sequences recruiting ribosomes. Because the short *mCherry* isoform is encoded in the *mCherry* gene sequence, it is highly likely that other prokaryotes also produce the short *mCherry* isoform. In addition, depending on the codon usage of the N-terminal extension, the short *mCherry* expression may be stronger or adapted to other organisms (as demonstrated by *mCherry* with SD-deoptimized) (Figure 1). We found that the short *mCherry* isoform (V1) was functional in *Vibrio natriegens* and *Pseudomonas putida* (Supplementary Figures S5 and S6), and another group had found the *mCherry* ATIS occurring in *Mycoplasma* (Carroll et al., 2014). This demonstrates that the *mCherry* defect occurs across a wide range of bacteria.

**FIGURE 4:**
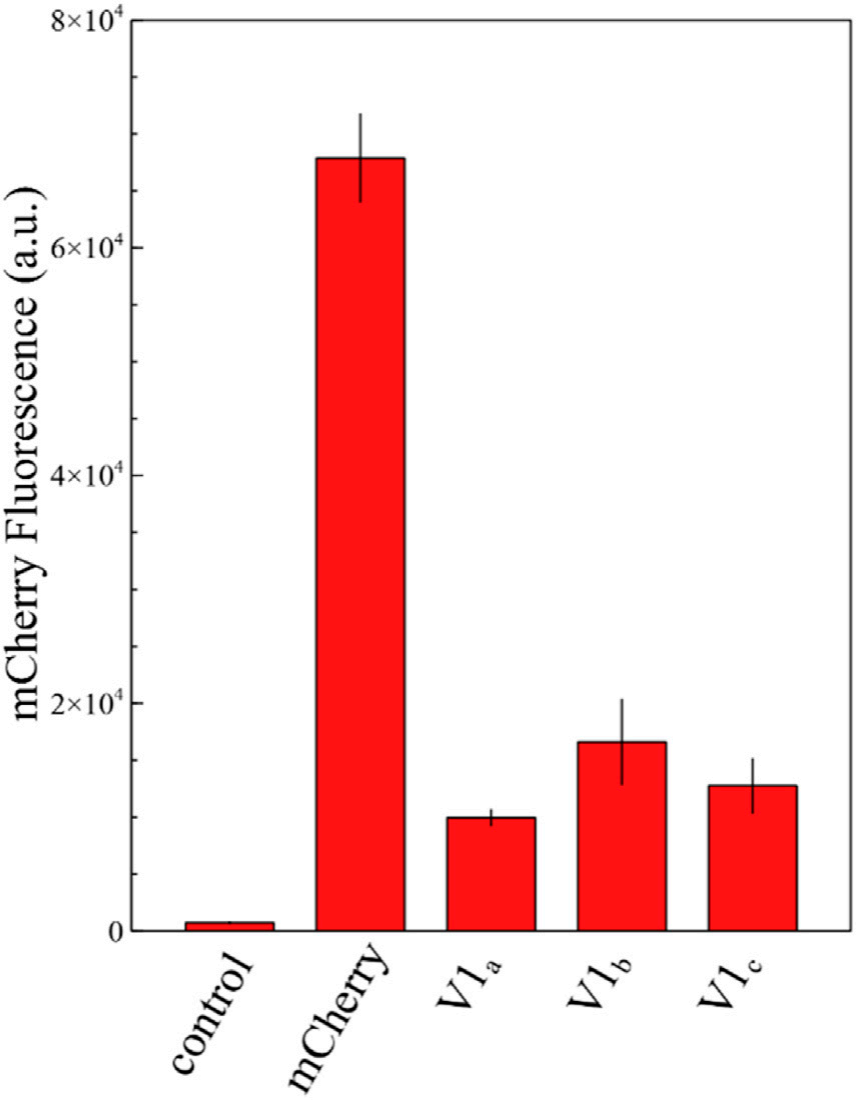
Fluorescence measurements of *mCherry* and its shorter isoforms V1 in the yeast *Saccharomyces cerevisiae*. The graph shows the fluorescence level of a *S. cerevisiae* strain carrying the yGPRA plasmid expressing the original *mCherry* sequence under the control of the TEF1 promoter and three independent yeast transformants carrying the mutated AUG to UAG original start codon of *mCherry*. The short *mCherry* isoform creates in average a 19% background fluorescence compared with the original sequence. The control shows the background fluorescence of the *S. cerevisiae* uracil-deficient strain. Each measurement was performed in biological triplicates.

Translation initiation significantly differs between prokaryotes and eukaryotes. Bacteria mainly rely on the annealing of 16 S ribosomal RNA with the SD sequence and mRNA structures surrounding the start codon, whereas eukaryotes lack this type of interaction with the mRNA transcript. Instead, the small ribosomal subunit (40 S), charged with tRNA_Met,i_, recognizes an AUG start codon through a scanning mechanism of codons within the 5′UTR of mRNA strands (Sonenberg and Hinnebusch, 2009; Hinnebusch, 2014). The nucleotide context around the start codon also plays a role in initiating translation, favoring specific nucleotide motifs, sequence named after Kozak who first identified it (Kozak, 1986). The consensus yeast Kozak sequence was determined as (A/U)A (A/CA (A/C)A AUG UC(U/C) (Hamilton et al., 1986); however, this sequence varies a lot both within and across organisms (Nakagawa et al., 2008; Cuperus et al., 2017). To assess if the short *mCherry* isoform is also produced in eukaryotes, we replaced the AUG start codon with the UAG stop codon from a *S. cerevisiae* codon-optimized *mCherry*. In addition, we modified the codon usage surrounding M10 to GAAGAAGACAAC AUG GCC to resemble the optimal yeast Kozak sequence described by de Boer et al. (2020) and Hamilton et al. (1986). We demonstrated that this *mCherry* sequence provides in average a 19% background fluorescence compared with the original sequence (Figure 4; Supplementary Figure S7)*.* It conclusively proves that the eukaryotic scanning mechanism of translation initiation allows the production of the short *mCherry* isoform. The nucleotide context surrounding the start codon may influence the level of translation in different eukaryotic cells. However, the ability of yeast to translate the short *mCherry* isoform raises concerns regarding studies using *mCherry* as a fluorescent marker across eukaryote species.

### Solutions deployed to circumvent the defect in *mCherry*

**FIGURE 5:**
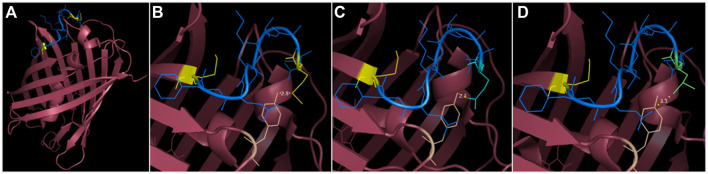
3D structure visualization of *mCherry* and the substitutions provided to abolish the short isoform. **(A)** the segment between M10 and M17 (shown in blue, methionine in yellow) precedes the first ß-sheet but provides indispensable stabilization to the protein (as *mCherry* V2 is not functional). The eGFP segment preceding M10 is not present on any PDB files of *mCherry*, potentially because of a low resolution, due to mixed isoform population. **(B)** M10 (yellow) stabilizes *mCherry* by interacting with Y43 (wheat color). **(C)** M10Q substitution (in cyan) occupies the same volume as methionine and can produce an H-bond with Y43. **(D)** M10L substitution (in lime green) also mimics the steric occupancy of methionine and can weakly interact with Y43 through Van der Wall’s interaction.

To provide a version of *mCherry* that is usable for gene expression and protein localization without conferring background expression, we tested different solutions in the context of fusion protein. The first solutions consisted in a substitution of the problem-causing M10 to glutamine or leucine, which aims to preserve the structural properties of the protein (Figure 5). As M10 stabilizes *mCherry* by interacting with the tyrosine residue Y43, the substitutions of M10 with glutamine or leucine allow similar stabilization through the formation of an H-bond with Y43 or with Van der Walls interactions, respectively, while occupying an equivalent steric space as methionine. The second solution was simply to use *mCherry* V1 as a reporter as it resembles *mRFP1.1*.

**FIGURE 6:**
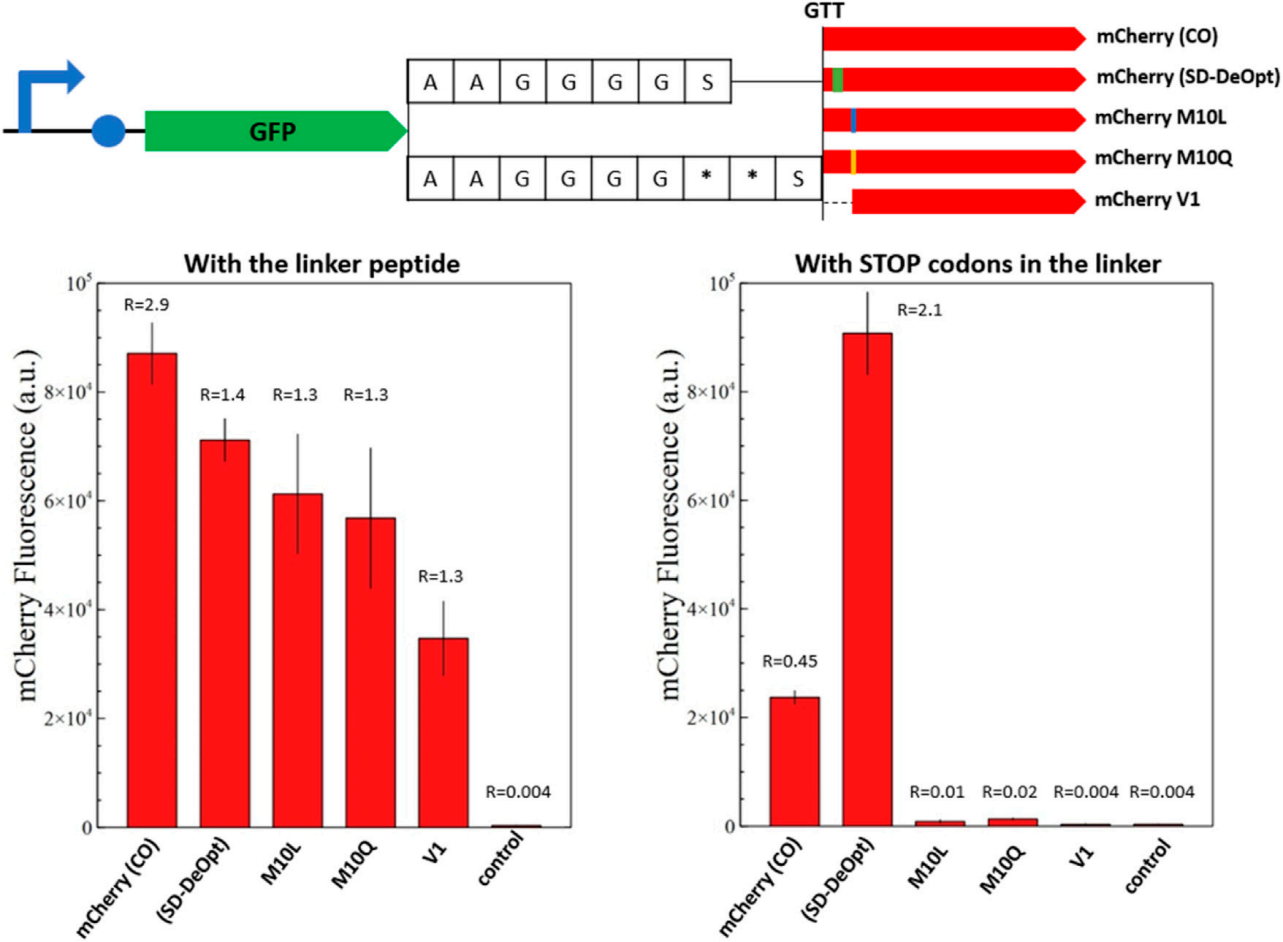
Fluorescence measurements of *mCherry* and its modified versions in the context of C-terminal fusion. The overview of the genetic constructs is presented (top). The versions of *mCherry* are fused to the C-terminal end of sfGFP with a standard alanine/glycine linker or with the linker containing two stop codons. The red fluorescence of the fusion proteins with the alanine/glycine linker is presented on the left-hand side and with stop codons on the right-hand side. The graphs show the *in vivo* fluorescence of the different *mCherry* versions in *E. coli* normalized by OD_600_. Four biological replicates were made for each *mCherry* version, and the respective standard deviation is represented with black error bars. A plasmid expressing only sfGFP is used as a negative control. The ratio between sfGFP and *mCherry* fluorescence is indicated above the corresponding histogram bar (from Supplementary Figure S4). Just *mCherry* (CO) and the “Shine–Dalgarno-deoptimized” version show background fluorescence with the linker containing stop codons. The M10 substitutions prove successful in abolishing the short *mCherry* isoform in the context of C-terminal fusion. Likewise, the short *mCherry* V1 does not show background fluorescence but appears to be less efficient in C-terminal fusions.

Fusion proteins composed of sfGFP fused with C-terminal engineered versions of *mCherry* were constructed to assess the performance of each solution. First, sfGFP and *mCherry* versions were linked with an alanine/glycine linker to estimate the C-terminal fusion properties of the engineered *mCherry* versions (Figure 6). Second, the background expression due to the short isoform was assessed by placing two stop codons in the linker peptide right upstream the *mCherry* versions.

In both cases, GFP is used as an internal standard of gene expression to compare the *mCherry* production as fusion or discrete protein between samples. The fusion proteins composed of the mutated amino acids in position 10 or the V1 isoform display an *mCherry*/GFP ratio of 1.3, which constitutes a reference under the assumption of an equimolar production of mCherry and GFP because they are expressed as a fusion protein (Figure 6). The inclusion of stop codons in the protein linker confirms this assumption, as none of these constructs displays *mCherry* fluorescence when the full-length fusion protein is not produced.

In the context of fusion protein, the original *mCherry*-(CO) shows a nearly twofold increase in *mCherry* abundance that can be attributed to the short isoform; however, this was not clearly detected for the *mCherry*-(DeOpt) version that should present the same characteristics (Figure 6). This may suggest a higher proportion of V1 isoform and truncated translation of sfGFP-*mCherry* fusions because ribosomal entry at the internal SD sequence of *mCherry-*(DeOpt) may cause upstream-translating ribosomes to fall off at the protein junction with *mCherry*. The two M10-mutated versions of *mCherry* present the same expression intensities as the original protein sequences in the fusion context; whereas the fluorescence of the short *mCherry* V1 isoform is slightly lower but still reasonable (Figure 6). This demonstrates that all solutions provided to resolve the *mCherry* issue are functional in a fusion protein context.

For the fusion proteins containing stop codons, the original *mCherry*-(CO) sequence and the “SD-deoptimized” version present a background fluorescence due to the production of the short isoform, which is amplified in the case of “SD-deoptimized” due to higher ribosomal entry on the 5′end of the *mCherry* sequence as its internal SD sequence favors V1 isoform translation. On the contrary, the M10 substitutions and V1 isoform do not show any fluorescence, which proves that these solutions successfully abolish the production of the short *mCherry* isoform in the context of C-terminal fusion (Figure 6). Likewise, the short *mCherry* V1 did not show any background fluorescence but appeared to be less efficient in C-terminal fusions, as reported for *mRFP1.1* (Shaner et al., 2004).

Subtle changes in the amino acid sequence of fluorescent proteins can cause changes in their spectral properties as well as their brightness and maturation (Arpino et al., 2014). The removal of the N-terminal sequence before M10, or the substitutions of M10 by leucine and glutamine, seems to affect the brightness and/or maturation of the protein and potentially its ability to be used as soluble fusion partner. However, we detected no changes in the excitation/emission spectra for the different variants (Supplementary figure S8). Further investigation and engineering are necessary to recover the properties of *mCherry* without generating the short protein isoform that creates background fluorescence.

## Conclusion

The unexpected performance of *mCherry* as a reporter in our gene expression experiments led to the discovery of a short functional isoform of *mCherry*. The expression of this short isoform affects the outcome of gene expression and protein localization studies. The defect originates from a methionine residue in position 10 that is preceded by an SD-like sequence. We showed that the mutation of this residue to leucine or glutamine abolishes the production of the short isoform while conserving N-terminal fusion properties.

Our findings indicate that a large proportion of the DsRed-derived proteins are affected by the production of the short isoform due to the presence of a problem-causing linker sequence (MVSKGEE-NNMA). In addition, we identified the presence of this linker in the bright-green fluorescent protein mNeonGreen (Shaner et al., 2013). We would like to highlight the distinct possibility that the production of short functional isoforms will also affect other fluorescent proteins. Indeed, the presence of methionine residues in the first 10–20 amino acids, which may act as an alternative translation start sites, is widespread among fluorescent proteins. For example, mTFP1 (Ai et al., 2006), KillerRed (Bulina et al., 2006), Dronpa (Ando et al., 2004), mEosEM (Fu et al., 2020), mKelly2 (Wannier et al., 2018), mGinger2 (Wannier et al., 2018), and their derivatives could be affected by the production of a short isoform as observed for *mCherry*.

In addition, we showed that the dual-isoform issue affects various prokaryotes as well as eukaryotes despite having distinct translation initiation mechanisms. On the one hand, prokaryotes produce the short isoform of *mCherry* due to the affinity of the 16 s ribosomal RNA and the internal SD sequence as well as the recognition of the downstream M10 methionine. On the other hand, the eukaryotic 40 S ribosome scans the 5′UTR region of the mRNA strand to find translation start sites. The nucleotide features of the 5′UTR sequence, such as the presence of an SD or a Kozak sequence, low mRNA structures, and presence of the alternative start codon, enable the translation of the short *mCherry* isoform both in prokaryotes and eukaryotes.

In this study, we showed that the presence of an SD-like sequence upstream M10 dramatically increases the expression of the short *mCherry* isoform. Hence, an unfortunate nucleotide combination upstream M10 due to a certain codon usage can promote translation of the short *mCherry* isoform and lead to a higher background fluorescence. We recommend using the mutated versions of *mCherry*, M10Q or M10L, to avoid any expression background related to alternative translation start sites.

We demonstrate that M10 substitution abolishes the expression of the short protein isoform. This solution should be investigated for all affected DsRed-derived proteins to ensure their accuracy and reliability as reporters. Moreover, we suggest that similar actions might be necessary for other fluorescent protein families. This work raises concerns on the outcome of studies that employed fluorescent proteins that possess an inherent background expression.

## Materials and methods

### Materials

Experiments were performed in *E. coli* DH5-α (NEB), grown in LB-Lennox (Oxoid) (10-g/L casein peptone, 5-g/L yeast extract, 5-g/L NaCl with additional 15-g/L agar for plates) supplemented with 100-µL/mL ampicillin (Sigma-Aldrich). PCR amplifications were performed using Q5 polymerase (NEB). All other necessary enzymes were also purchased from NEB. Primers were ordered from Eurofins Genomics – Sigma-Aldrich. Plasmids and PCR products were purified using the QIAprep Plasmid Miniprep Kit and QiaQuick PCR Purification Kit, respectively (Qiagen). Plasmid Sanger sequencing was performed by Eurofins Genomics. The *E. coli* codon-optimized sequence of *mCherry* was a gift from Yanina R. Sevastsyanovich (University of Birmingham).

### Cloning and strain engineering

A pUC19 backbone containing a constitutive promoter/5′-UTR expressing *sfGFP*, generated in a previous study (Lale et al., 1101), was used as template for cloning the different versions of *mCherry*. The backbone and the different versions of *mCherry* were amplified by PCR using the respective primers presented in Supplementary Table S1. To test for short isoforms, *sfGFP* was replaced by the codon-optimized *mCherry* gene or its shorter versions (V1, V2, and V3) *via* Golden Gate assembly (Engler et al., 2008). To build fusion constructs, the different *mCherry* versions were fused downstream *sfGFP* using the Golden Gate assembly.

The Golden Gate assembly mixture was chemically transformed into competent *E. coli* by heat shock (45 s at 42°C). Cells were placed on LB plates containing 100-µg/mL ampicillin and grown overnight at 37°C. Positive clones were grown in 5-mL LB supplemented with 100-µg/mL ampicillin, and their respective plasmids were purified. DNA sequences were confirmed by Sanger sequencing.

For *S. cerevisiae*, we used the yGPRA plasmid kindly provided by Regev et al. (de Boer et al., 2020). In brief, it contains a yeast codon-optimized *mCherry* gene expressed by the TEF1 promoter. The AUG start codon was replaced by an UAG stop codon, and the codon usage surrounding M10 was modified to GAAGAAGACAAC AUG GCC to resemble yeast Kozak sequence *via* PCR using primers presented in Supplementary Table S1. The PCR products were subject to overnight DpnI digestion at 37°C, subsequently purified, assembled using the Gibson assembly, and transformed into *E. coli* Dh5α. Correct assembly was confirmed by restriction digest with the BbsI and speI enzymes of the plasmid as well as sequencing (Eurofins Genomics). The original yGPRA plasmid and the modified yGPRA_*mCherry*_V1 plasmid were transformed into the uracil-deficient *S. cerevisiae* strain using the previously described electroporation method (Benatuil et al., 2010). The transformants were selected onto SD–UT media (6.7-g/L yeast nitrogen base without amino acids, 1.6-g/L yeast synthetic dropout medium supplement without uracil, and 20-g/L agar).

### Fluorescence measurements

Each *E. coli* strain bearing a given plasmid was inoculated into LB supplemented with 100-µg/mL ampicillin on 96-well plates and incubated overnight at 37°C with 800-rpm agitation in a Multitron Pro plate-shaking incubator (Infors HT). For yeast, positive clones for both yGPRA and yGPRA_*mCherry*_V1 plasmids were cultivated in triplicates in 5-mL SD–UT media. The uracil-deficient *S. cerevisiae* strain was cultivated in triplicates in 5-mL YPD media, collected *via* centrifugation at 5,000 rpm, and then washed with SD–UT media before fluorescence measurements. Each sample (100 µL) was used for fluorescence measurements. Fluorescence was measured using an Infinite M200 Pro TECAN fluorimeter (Noax Lab AS). The excitation/emission wavelengths were 488/525 nm for GFP (gain 67) and 576/610 nm for *mCherry* (gain 97 for *E. coli* and 163 for yeast). Fluorescence intensity was normalized by OD_600_ of the corresponding well.

### Fluorescence microscopy

Cell cultures of *E. coli* and *S. cerevisiae* carrying the respective plasmids expressing *mCherry* and the V1 isoform were placed between glass slides for the microscopic analysis. An inverted fluorescence microscope (Zeiss Axio Observer. Z1, 2.3.64.0) with a ×20 air objective (NA 0.8) was used to detect *mCherry* and was localized within the cells using the bright field filter. Image processing was performed using the Zeiss image analysis software (2.3.64.0). 

### Bioinformatics analysis

The amino acid sequence of *mCherry*-(CO) was used as template to perform preliminary protein BLAST searches (blast.ncbi.nlm.nih.gov/Blast.cgi). Phylogeny of red fluorescent proteins was consulted on FPbase [27] (fpbase.org).

### Proteomics analysis

The strain carrying the constitutively expressed *mCherry* V1 was grown overnight in 50 mL of LB media supplemented with 100-µg/mL ampicillin at 37°C, 225 rpm. Cells were harvested by centrifugation (5,000 rpm, 5 min) and resuspended in lysis buffer (50 mM Tris-HCl, 50 mM NaCl, 0.05% Triton X-100, pH 8.0) supplemented with 1 tablet EDTA-free cOmplete ULTRA protease inhibitor (Roche). Cell debris were eliminated *via* centrifugation (7,500 rpm, 15 min), and the soluble fraction was collected.

### Sample preparation for LC-MS—protein digestion

Two samples of 200-µL cell lysate each were separated for LC-MS analysis, one to be digested by trypsin alone and the other by both trypsin and Lys-C. Soluble proteins were precipitated by methanol/chloroform/water precipitation. In brief, 800-µL methanol was added to the sample, followed by the addition of 200-µL chloroform and vortexing. After the addition of 600-µL ultrapure water (18 MΩ), samples were thoroughly mixed by vortexing and centrifuged at 16000 g for 2 min. The upper layer was discarded without disturbing the protein layer, and further 800-µL methanol was added, followed by vortexing and centrifugation as above. After removing the supernatant, the protein pellet was air-dried for 10 min and was then reconstituted in 150 µL of 50-mM ammonium bicarbonate (BioUltra; Sigma-Aldrich, Germany). Next, the proteins were reduced with 1.5 µL of DTT (Sigma-Aldrich, Canada) for 20 min at 70°C, brought back to room temperature, and then alkylated using 6 µm of iodoacetamide (BioUltra; Sigma-Aldrich, United States) in the dark at room temperature for 30 min. Excess iodoacetamide was quenched by adding 3.5 µm of DTT (Sigma-Aldrich, Canada) and incubating in the dark for 20 min at room temperature. At last, the proteins were digested with endoproteinase at 37°C overnight, one sample digested with 1.25-µg trypsin and the other by 1.25-µg trypsin and 1.25-µg Lys-C. After overnight digestion, 5 µL of formic acid was added to each sample, and then the peptides were dried in a vacuum concentrator at 60°C.

### Sample preparation for LC-MS—peptide desalting

The samples were resuspended in 100-µL 0.1% formic acid and desalted in C18 stage tip columns, unless otherwise specified. The chemicals were of Optima grade obtained from Fisher Chemicals, and centrifugations were performed at 2000 g. In brief, stage tip columns consisting of three C18 plugs (Empore C18 47 mm SPE Disks, 3 M, United States) were made and activated using 50-µL methanol *via* centrifugation for 2 min; then, methanol activation was repeated. Then, the stage tip column was equilibrated with 60 µL of 0.1% formic acid in water *via* centrifugation for 2 min; then, the equilibration was repeated. The peptide samples were centrifuged (16000 g for 25 min), supernatants were loaded onto stage tip columns and centrifuged for 4 min, and flow-through solutions were reloaded to stage tip columns. The stage tip columns were washed with 60 µL 0.1% formic acid and centrifuged for 2 min; the wash was then repeated. The peptides were then eluted from the stage tip column using 40 µL of 70% acetonitrile, washed with 0.1% formic acid, and then centrifuged for 2 min; the elution step was then repeated. At last, desalted peptides were dried in a vacuum concentrator at 60°C and stored at −20°C until LC-MS analysis.

### LC-MS analysis

Dried peptides were reconstituted in 50 µL of 0.1% formic acid in water and shaken at 6°C at 900 rpm for 1.5 h. The samples were centrifuged at 16000 g for 10 min, and 40-µL supernatants were transferred to MS vials for LC-MS analysis. LC-MS analysis was performed on an EASY-nLC 1200 UPLC system (Thermo Fisher Scientific) interfaced with a Q Exactive mass spectrometer (Thermo Fisher Scientific) *via* a Nanospray Flex ion source (Thermo Fisher Scientific). Peptides were injected onto an Acclaim PepMap100 C18 trap column (75 µm i. d., 2-cm long, 3 µm, 100 Å, Thermo Fisher Scientific) and further separated on an Acclaim PepMap100 C18 analytical column (75 µm i. d., 50-cm long, 2 µm, 100 Å, Thermo Fisher Scientific) using a 180-min multistep gradient (150 min 2%–40% B, 15 min 40%–100% B, 15 min at 100% B, where B is 0.1% formic acid and 80% CH_3_CN and A is 0.1% formic acid) at 250-nL/min flow. The peptides were analyzed in the positive ion mode under data-dependent acquisition using the following parameters: electrospray voltage, 1.9 kV; HCD fragmentation with normalized collision energy, 28. Each MS scan (200–2000 m/z, 2-m/z isolation width, profile) was acquired at a resolution of 70,000 FWHM in the Orbitrap analyzer, followed by MS/MS scans at a resolution of 17,500 (2 m/z isolation width, profile) triggered for the 12 most intense ions, with a 30-s dynamic exclusion, and analysis using the Orbitrap analyzer. Charge exclusion was set to unassigned, 1, and >4.

### Processing of LC-MS data

The proteins were identified by processing the LC-MS data using Thermo Fisher Scientific Proteome Discoverer (Thermo Fisher Scientific) version 2.5. The following search parameters were used: enzyme specified as trypsin with maximum two missed cleavages allowed; acetylation of protein N-terminus with methionine loss, oxidation of methionine, and deamidation of asparagine/glutamine were considered as dynamic and carbamidomethylation of cysteine as static posttranslational modifications; precursor mass tolerance of 10 parts per million with a fragment mass tolerance of 0.02 Da. Sequest HT node was used to query the raw files against sequences for *mCherry* (original and short); *E. coli* (strain K-12) proteins were downloaded from UniProt (www.uniprot.org/proteomes/UP000000625) in September 2020 and a common LC-MS contaminants database. For downstream analysis of this peptide-spectrum matches (PSMs), for protein and peptide identifications the PSM FDR was set to 1% and as high and 5% as medium confidence; thus, only unique peptides with these confidence thresholds were used for the final protein group identification and for the labeling of the confidence level, respectively.

### Protein 3D structure modeling

The 3D structure modeling of *mCherry* was performed using the software PyMOL and the PDB file 2H5Q. In all *mCherry* PDB files (2H5Q, 6YLM, 6IR1 and 2, 6MZ3, 4ZIN), the first eight amino acids were unmodeled, residues 9 and 10 were computationally added, but M10 was mismodeled. We corrected residue 10 to model methionine. Then, we changed M10 into glutamine and leucine on the 3D structure with PyMOL. Rotamers with 2–3 Å proximity with Y43 are shown to model their interactions.

## Data Availability

The proteomics data have been uploaded onto the ProteomeXchange database (project accession number: PXD032954).

## Footnotes

* The mathematician Gottlob Frege dedicated his life to demonstrating that mathematics was reducible to logic. In June 1901, his ambitions were shattered by a letter from Bertrand Russell that pointed out a contradiction to his fundamental assumption that became famously known as “Russell’s paradox” (Chudakov et al., 2010). Russell’s paradox states the following: Consider *R* the set of all sets that do not contain themselves as members; if *R* is not a member of itself, then by definition, it is a member of itself, and reciprocally.
