# Supplementary material for: mCherry contains a fluorescent protein isoform that interferes with its reporter function
Source: Front Bioeng Biotechnol. 2022 Aug 9;10:892138. doi: 10.3389/fbioe.2022.892138 (PMC9395592; doi:10.3389/fbioe.2022.892138)
Supplement: Supplementary file 1 [file DataSheet1.PDF]

mCherry contains a fluorescent protein isoform that interferes with its reporter function

Authors

Maxime Fages-Lartaud[1], Lisa Tietze[1], Florence Elie[1], Rahmi Lale[1], Martin Frank Hohmann-Marriott[1,2]

Affiliation

[1]Department of Biotechnology, Norwegian University of Science and Technology, Trondheim, N-7491, Norway.

[2]United Scientists CORE (Limited), Dunedin 9016, Aotearoa - New Zealand.

Contact (corresponding author)

Email: martin.hohmann-marriott@ntnu.no

Supplemental Figures and Tables.

Supplemental Table S1. Primer list

**Name**	**sequence**	**comments**
MFL 641	GATTCAGGTCTCCCATAAGCCCTTTCTCCTTCCAGAGC	Backbone amplification including promoter/5'UTR for cloning of short *mCherry* versions
MFL 268	AGAGTAGGTCTCATCAACCGTCTTCAAGCGAGAACGC	
MFL 501	AGAGTTGGTCTCATATGGTTTCTAAAGGTGAAGAAGA	mCherry-(CO) forward
MFL 572	AGAGTTGGTCTCATATGGCTATCATCAAAGAATTTATGCG	mCherry-(V1) forward
MFL 644	AGAGTTGGTCTCATATGCGTTTCAAAGTTCACATGGAAGG	mCherry-(V2) forward
MFL 573	AGAGTTGGTCTCATATGGAAGGTTCTGTGAACGG	mCherry-(V3) forward
MFL 574	CTAGTAGGTCTCATTGATTATTTATACAGTTCGTCCATACCGC	mCherry reverse for all versions
MFL 690	AGAGTAGGTCTCAGCTTCCGCCTCCGCCTGCTGCTTTATACAGTTCGTCCATACCGTGG	Backbone amplification including the constitutively expressed sfGFP for fusion protein with mCherry with and wihtout Stop codons (each coupled with MFL 268)
MFL 691	AGAGTAGGTCTCAGCTTTATTATCCGCCTCCGCCTGCTGCTTTATACAGTTCGTCCATACCGTGG	
MFL 692	AGAGTAGGTCTCAAAGCGTTTCTAAAGGTGAAGAAGATAACATGGCTATCATC	mCherry-(CO) forward for fusion construct
MFL 693	AGAGTAGGTCTCAAAGCGTTTCTAAAGGAGAGGAGGATAACATGGCTATCATC	mCherry-(SD-DeOpt) forward for fusion construct
MFL 694	AGAGTAGGTCTCAAAGCGCTATCATCAAAGAATTTATGCGTTTC	mCherry-(V1) forward for fusion construct
MFL 695	AGAGTAGGTCTCAAAGCGTTTCTAAAGGAGAGGAGGATAACCTGGCTATCATC	mCherry-(M10L) forward for fusion construct
MFL 696	AGAGTAGGTCTCAAAGCGTTTCTAAAGGAGAGGAGGATAACCAGGCTATCATC	mCherry-(M10G) forward for fusion construct
MFL 851	ACTAAACACTAGTACCTAGGTGAGCAAGGGCGAAGAAGACAACATG	yeast mCherry V1 for gibson assembly - reverse
MFL 877	TCGCCCTTGCTCACCTAGGTACTAGTGTTTAGT	yeast mCherry V1 for gibson assembly - forward
MFL 865	TTGTCTGTGTAGAAAACCACACACGAAAATCC	yGPRA for gibson assembly - forward
MFL 866	GGATTTTCGTGTGTGGTTTTCTACACAGACAA	yGPRA for gibson assembly - reverse

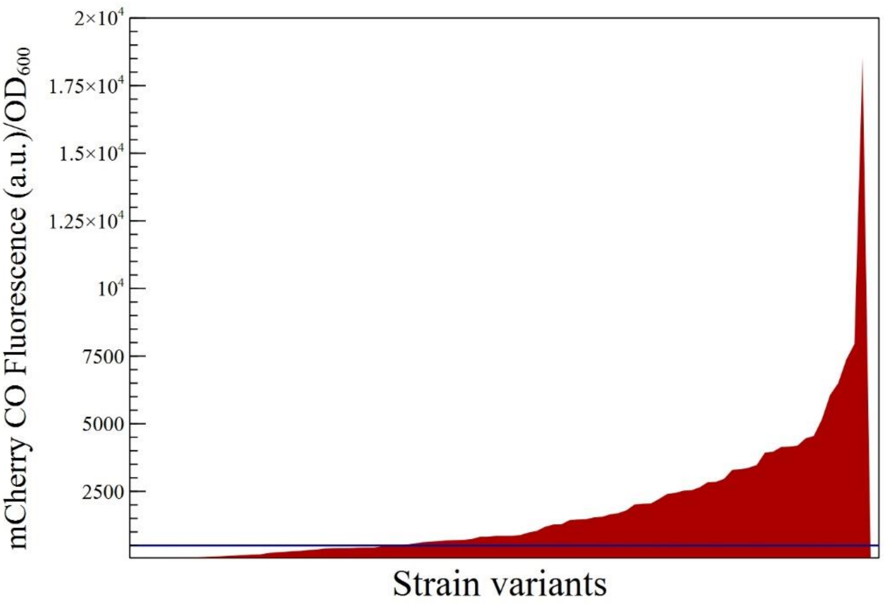

***Supplemental Figure S1*. Quantification of fluorescent *E. coli* strains carrying the 200N random library in front of the original *mCherry* gene.** The 200N library was cloned in front of the original mCherry (CO) and random clones were grown for fluorescence measurement. The number of positives clones obtained with *mCherry* exceeds significantly the usual 30-40% efficiency of the method. Here, the significance threshold (blue line) indicates that 65% of clones express mCherry.

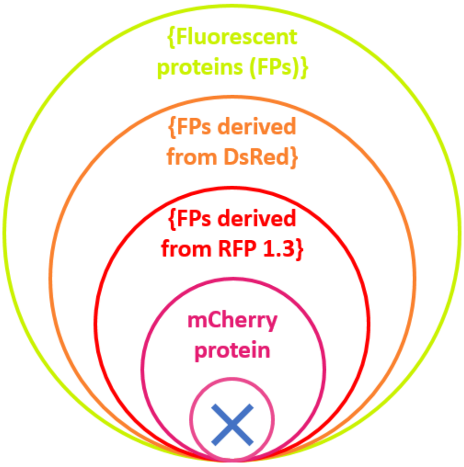

Supplemental Figure S2. **Representation of Russell's paradox applied to mCherry.** Each circle represents a set of fluorescent proteins that contains a smaller set. The mCherry protein is supposedly the smallest set. However, the circle with a cross represents Russell's paradox in the form of a short mCherry isoform contained in itself.

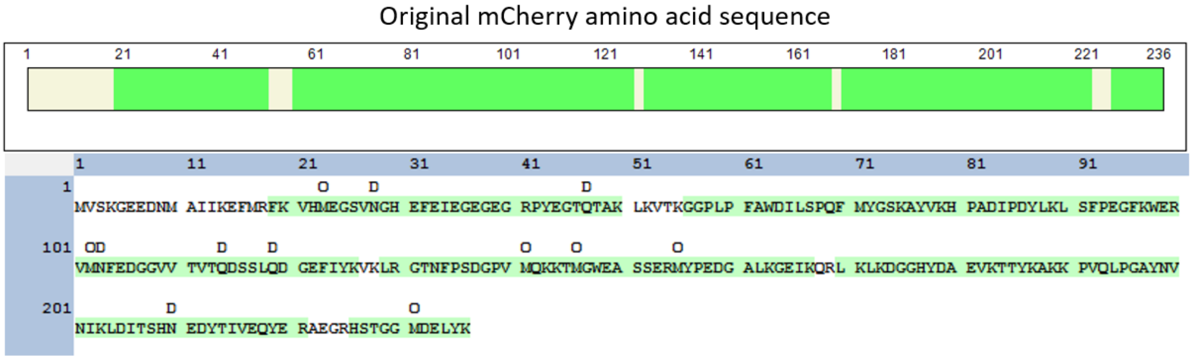

Supplemental Figure S3. **Peptide coverage of the mCherry V1 fluorescent protein found by proteomics.** Peptides identified by LC-MS analysis are displayed in green. The main initial fragment from G5 to K14 is absent, which confirms that the fluorescent isoform of mCherry lacks the N-terminal region. The fragment between M10 and M17 (difference between mCherry V1 and V2) was not detected due to the the cleavage site K14. However, mCherry V2 did not show fluorescence, thus mCherry V1 is the only short functional isoform.

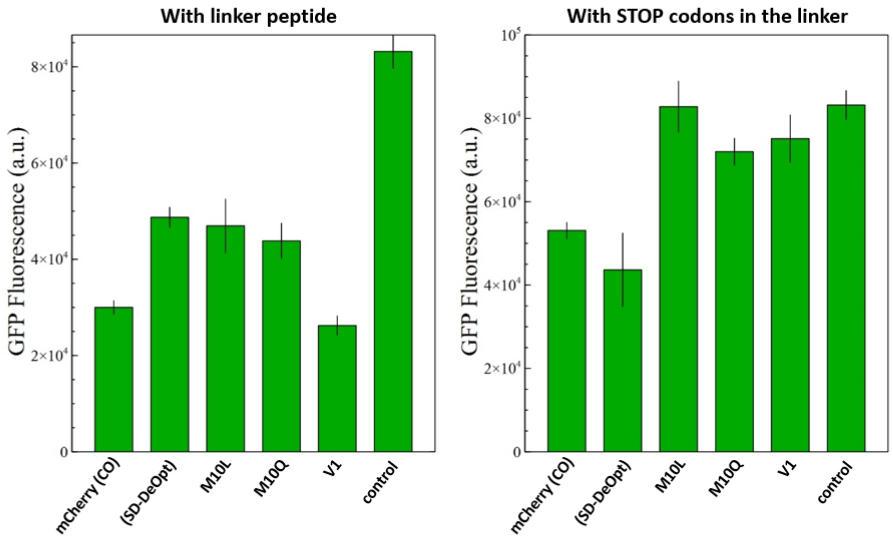

Supplemental Figure S4. **Fluorescence measurements of sfGFP from the fusions with the different versions of mCherry.** The sfGFP fluorescence was used to normalize the mCherry fluorescence and provide a relative ratio for comparison between mCherry versions (in *Figure 5*). A plasmid expressing only sfGFP is used as negative control.

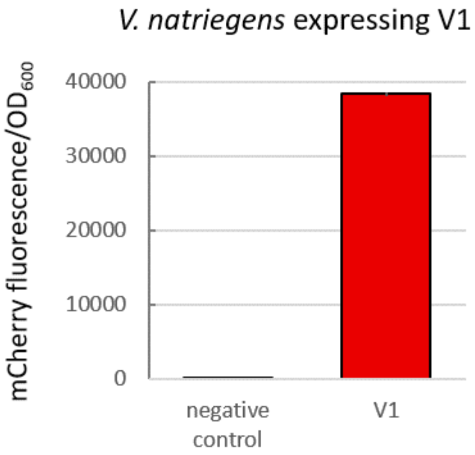

Supplemental Figure S5. **Fluorescence measurement of the short mCherry isoform V1 in *V. natriegenes.*** The short *mCherry* version was cloned onto a pACYC backbone and constitutively expressed in *V. natriegens*. The short gene produced a functional protein detected by fluorescence.

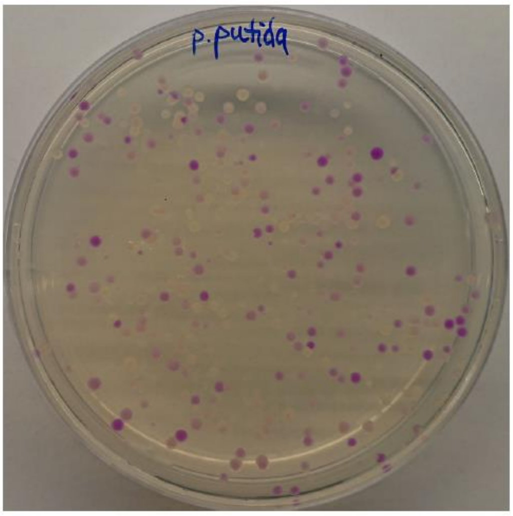

Supplemental Figure S6. **Photograph of *P. putida* expressing *mCherry* original gene with the 200N random DNA library.** The percentage of positive clones exceeds significantly the usual efficiency of the method, suggesting that the ATIS is also active in *P. putida*.

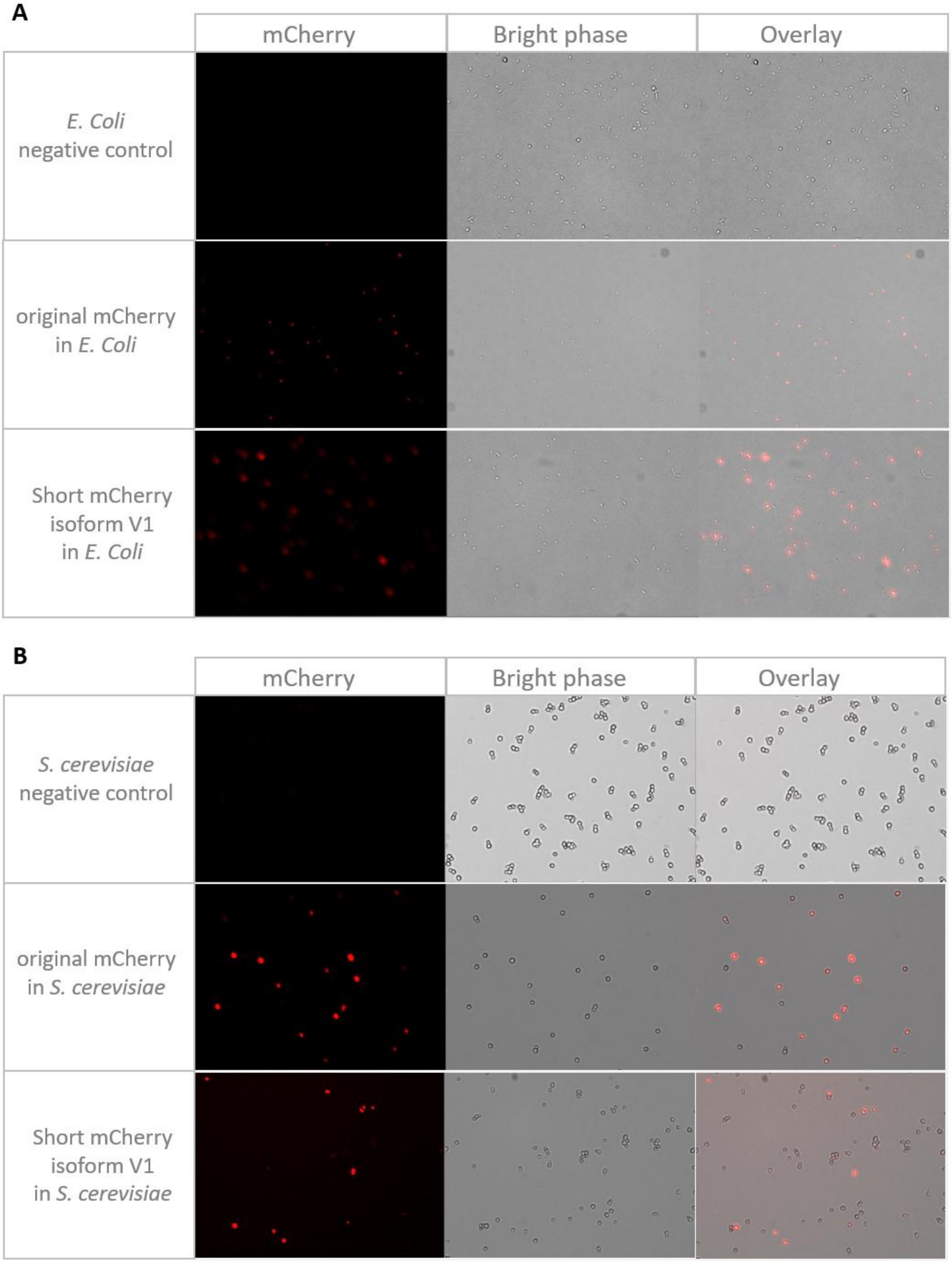

Supplemental Figure S7. **Fluorescence microscopy of (A) *E. coli* and (B) *S. cerevisiae* expressing either the original mCherry protein or the short isoform V1.**

A)

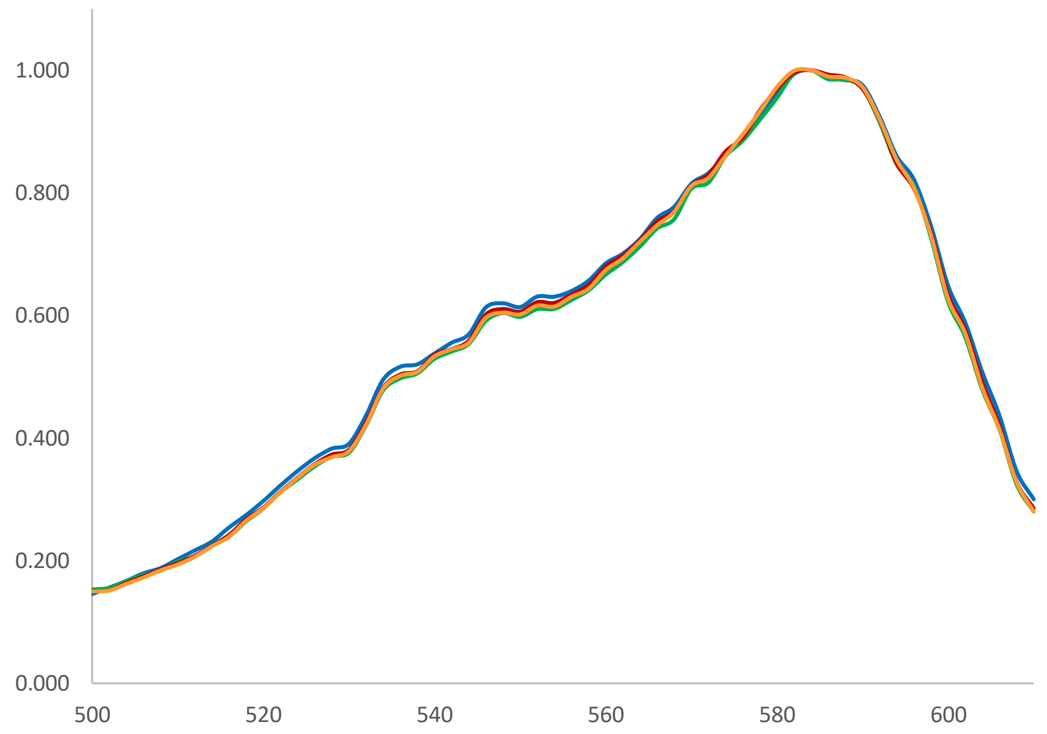

B)

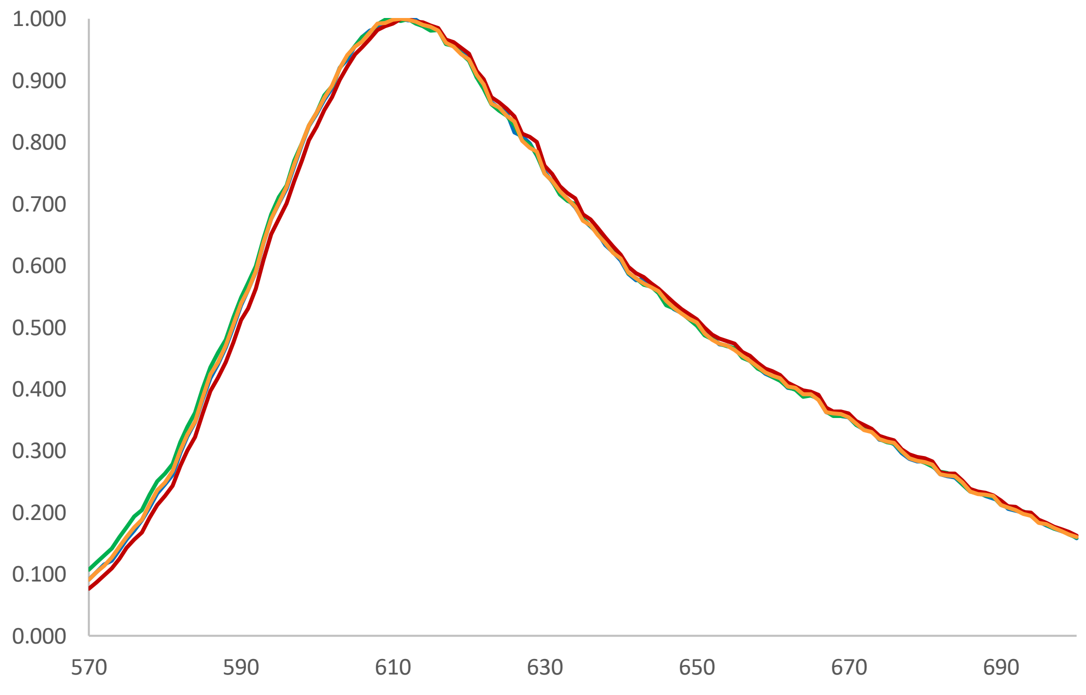

***Supplemental Figure S8.* Fluorescence spectra of the original mCherry protein, the short isoform V1 and the mutated versions M10Q and M10L.** (A) excitation spectra from 500 nm to 610 nm realized with a fixed emission wavelength of 645 nm, and (B) emission spectra from 570 nm to 700 nm with a fixed excitation wavelength of 525 nm for mCherry-CO (blue), V1 (green), M10Q (red), M10L (orange).
